# Effects of Emulsifier and Lubricant Formulation on Basalt Fiber Sizing Emulsions and Fiber Performance

**DOI:** 10.3390/polym18212645

**Published:** 2026-10-25

**Authors:** Jiajia Yuan, Junlan Li, Simin Zhuo, Jiahao Wu, Jianhui Wu, Like Zou, Yuanfang Deng, Chuan Lai

**Affiliations:** 1 Basalt Fiber and Composite Key Laboratory of Sichuan Province, School of Modern Industry for Basalt Fiber Composite Materials, Dazhou Key Laboratory of Advanced Technology for Fiber Materials, Sichuan University of Arts and Science, Dazhou 635000, China; 2 School of Chemistry and Environmental Engineering, Sichuan University of Science and Engineering, Zigong 643000, China; 3 Dazhou Market Supervision and Administration Bureau, Dazhou 635000, China

**Keywords:** basalt fiber, sizing, emulsifier, hydrophilic–lipophilic balance, orthogonal design

## Abstract

Basalt fibers are prone to friction-induced damage during drawing and processing, while sizing emulsion properties significantly affect coating uniformity and fiber protection. This study aimed to optimize basalt fiber sizing formulations by evaluating the effects of emulsifier and lubricant combinations on emulsion characteristics and fiber performance. An industrial sizing formulation was selected as the reference, and three nonionic emulsifiers, namely poly(ethylene glycol) dibasic acid diester (DAPE), poly(ethylene oxide)-poly(propylene oxide) block copolymer (L44) and sorbitan monolaurate (S20) with two lubricants including polyoxyethylene esters of saturated (M) and unsaturated fatty acids (N) were investigated using independent L9(3^3^) orthogonal experiments. A total of 27 formulations were prepared and evaluated based on solids content, viscosity, particle size, centrifugal stability, and surface tension. Six optimized formulations were further validated through fiber drawing tests. The results showed that sizing performance was controlled by the synergistic effects of emulsifier type, dosage, and lubricant ratio. Compared with the original sizing, the optimized formulations increased single-filament tensile strength by 4.46–20.82% and yarn breaking strength by 10.87–34.78%, with RA-1 (1.8% DAPE, 1.0% M, 1.6% N) exhibiting the best performance. After acid exposure, the optimized formulations maintained higher absolute yarn breaking strength than O-2, although relative strength retention varied only slightly among formulations. In contrast, alkaline exposure caused substantial strength loss, with retention rates of 50.94–53.70% and no significant differences among formulations.

## 1. Introduction

Basalt fiber (BF) is an environmentally favorable, high-performance inorganic fiber with attractive mechanical strength, chemical resistance, and thermal stability, and has found increasing interest in transportation, construction, energy, and polymer-matrix composite applications [1,2,3,4,5,6]. However, in the drawing of basalt fibers, rapid cooling and contraction give rise to a rather smooth surface, while the successive steps of continuous drawing, bundling, and post-processing tend to generate surface defects, fuzz, and broken filaments, ultimately impairing the mechanical performance of the final fibers [7,8,9,10]. A uniform and continuous surface layer with appropriate lubricity is therefore required during drawing to protect newly formed filaments and maintain stable production.

Sizing agents are an essential processing aid in continuous-fiber manufacture and generally contain film formers, coupling agents, lubricants, emulsifiers, pH regulators, and other additives [11,12,13,14,15]. For reinforcing BF sizing agents, the film former typically serves as the primary functional component and develops a continuous protective coating on the filament surface [16,17,18], promoting filament cohesion while regulating strand stiffness and flexibility. Lubricants provide wet and dry lubrication, reducing friction both between filaments and between the fibers and processing equipment [19,20], thereby improving strand integrity and processability. Coupling agents provide chemical functionality at the inorganic-fiber/organic-matrix interface and can facilitate subsequent interfacial bonding in composites [21,22,23,24]. Although emulsifiers are typically used at relatively low concentrations, they play an important role in dispersing hydrophobic components in the aqueous phase and regulating interfacial tension, droplet size, and emulsion stability [25,26,27]. Hence, understanding how the emulsifier type modifies the physicochemical behavior of sizing emulsions is important for formulation design and optimization.

The stability of sizing emulsions is governed by interfacial properties, formulation composition, and interactions between dispersed droplets [28,29,30,31]. In nonionic emulsifier systems, hydrated molecular chains provide steric barriers, but droplets may still aggregate, coalesce, or undergo Ostwald ripening during storage [32,33]. HLB is a useful starting point for emulsifier selection [34,35], but it cannot fully capture the effects of film formers, coupling agents, and lubricants in practical formulations. Emulsion viscosity is also relevant to handling, deposition, and sizing application [36,37]. Nevertheless, viscosity measurements under a single set of testing conditions are insufficient to fully characterize emulsion rheology. Further investigation is needed to examine the effects of the type of emulsifier, the emulsification dosage, and the proportion of lubricant on the physical and chemical properties of the emulsion as well as the final fiber performance.

Previous studies on BF sizing agents have largely focused on film-former selection and molecular design, as well as the formulation of coupling agents and lubricants [20,38,39,40,41,42,43]. By comparison, the combined effects of emulsifier selection and lubricant composition on emulsion properties and fiber performance remain less well understood. Lubricants commonly used in sizing formulations, including long-chain quaternary ammonium surfactants and polyoxyethylene ether- or ester-type nonionic surfactants, may also contribute to emulsification [44,45]. Excessive lubrication may interfere with coupling-agent performance, whereas insufficient lubrication can increase fuzz formation and abrasive damage during drawing. Therefore, emulsifier and lubricant content should be optimized together to balance emulsion formation, fiber protection, and processability without unnecessarily increasing the total surfactant content.

**Figure 1:**
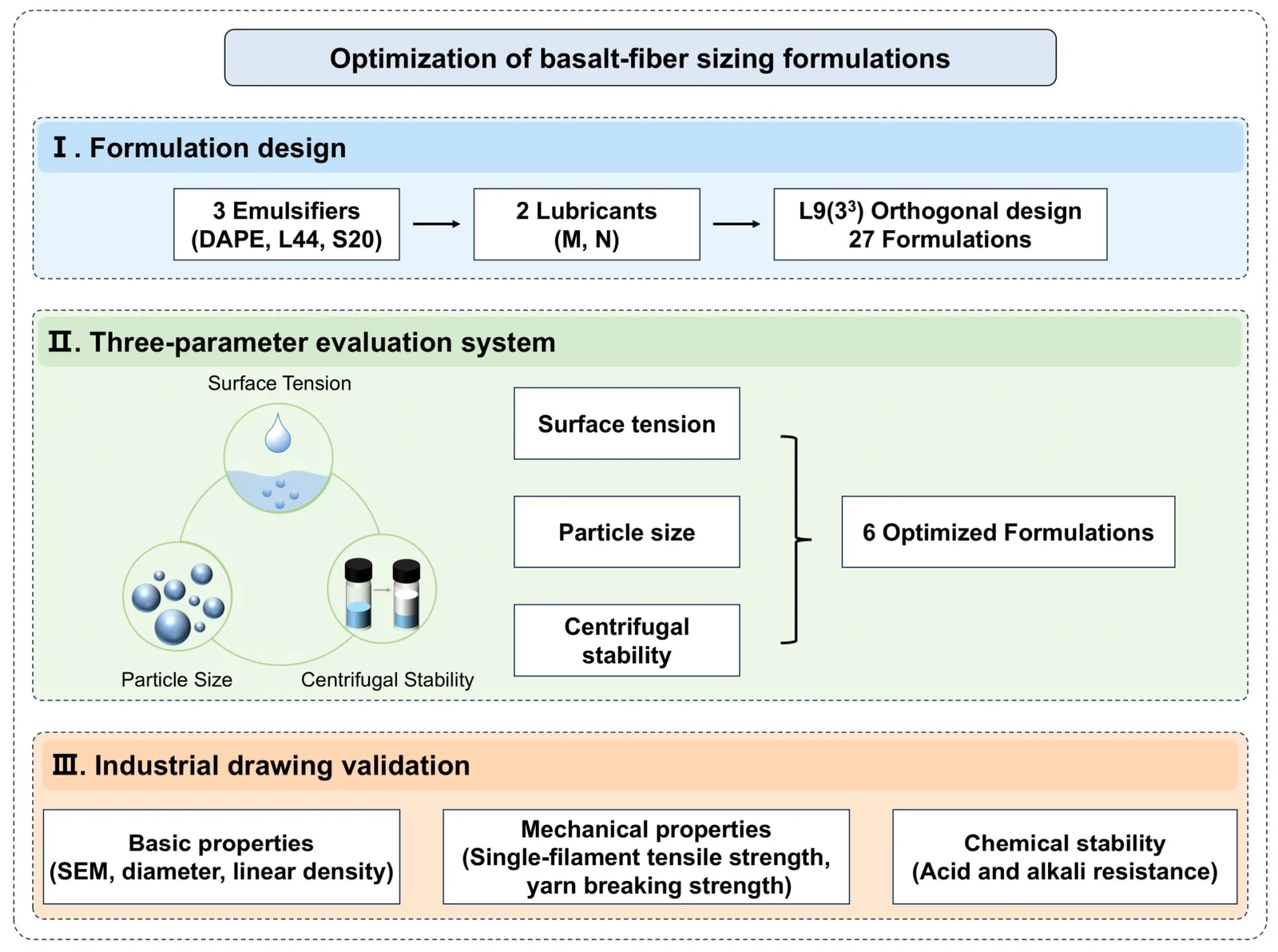
The optimization method of the sizing agent formula.

Based on the above considerations, this work investigates the combined effects of emulsifier type and emulsifier–lubricant ratio on the performance of a BF sizing system using a sizing formulation that has been industrially applied in basalt fiber production. As shown in Figure 1, three emulsifiers, namely DAPE, L44, and S20, are selected, and orthogonal experimental designs are established for each emulsifier series to collectively optimize the dosages of the emulsifier, lubricant M, and lubricant N. The resulting emulsions were characterized in terms of solids content, viscosity, particle size, centrifugal stability, and surface tension. Surface tension, particle size, and stability were then integrated into a three-parameter scoring scheme for the basic ranking of formulations within each emulsifier series. Selected formulations were finally applied directly in industrial fiber drawing under the same conditions, and the mechanical and corrosion-related properties of the resulting basalt fibers were assessed.

## 2. Materials and Methods

### 2.1. Materials and Sizing Agent Formulation

The baseline formulation was an industrial basalt fiber (BF) sizing emulsion used to produce reinforced unsaturated polyester resin (UPR) composites (Table 1). Its principal constituents included an unsaturated polyester film former and a water-soluble modified unsaturated epoxy auxiliary film former, both supplied by Changzhou Meikelai Composite Materials Co., Ltd. (Jiangsu, China); the silane coupling agent KH-570, supplied by Jiangxi Chenguang New Materials Co., Ltd. (Jiangxi, China); two lubricants, M and N, and the emulsifier DAPE, all supplied by Nantong Qianhe Chemical Co., Ltd. (Jiangsu, China); and deionized water prepared in the laboratory. Lubricant M was a polyoxyethylene ester of a saturated fatty acid, whereas lubricant N was a polyoxyethylene ester of an unsaturated fatty acid. Both lubricants had hydrophilic–lipophilic balance (HLB) values of approximately 12.

To generate emulsification systems with different interfacial characteristics, three nonionic emulsifiers were selected: DAPE (dibasic-acid polyoxyethylene ester, HLB = 16–17), L44 (poly(ethylene oxide)-poly(propylene oxide) block copolymer, HLB = 12), and S20 (sorbitan monolaurate, HLB = 8.6). Their HLB values were respectively higher than, close to, and lower than those of lubricants M and N (HLB ≈ 12). The amounts of the main film former, auxiliary film former, and KH-570 were kept constant. Emulsifier type and dosage and the M/N lubricant ratio were varied to determine how the emulsification–lubrication system influenced the sizing emulsion.

### 2.2. Preparation of Sizing Emulsions

KH-570 was hydrolyzed under acidic conditions before use. Deionized water at room temperature, corresponding to approximately 20–30 times the mass of KH-570, was adjusted to pH 3–4 with glacial acetic acid. KH-570 was then added slowly under vigorous stirring and allowed to hydrolyze for approximately 40–60 min until the initially turbid mixture became clear and no oily film was visible at the surface. The film was diluted with deionized water at 1–5 times its own mass. Lubricant M was dispersed in approximately 20 times its mass of deionized water at 50–70 °C until no flocculated material remained, while lubricant N was treated with approximately 5 times its mass of water at 50–90 °C until the flocs disappeared completely. DAPE was prediluted with approximately twice its mass of deionized water at room temperature. L44 and S20 were each dispersed in approximately 20 times their mass of deionized water and stirred at 50–70 °C for 60–90 min until no visible oily phase remained. The components were then added slowly to the film-former dispersion in the following order: lubricant M, DAPE (or L44/S20), auxiliary film former, and lubricant N. The hydrolyzed KH-570 was added subsequently, the formulation was brought to the target mass with deionized water, and the emulsion was mechanically stirred for at least 60 min before labeling and storage.

Prior to the orthogonal experiments, the emulsifier dosage range and the homogenization time of the sizing emulsion were investigated. As summarized in Table S1, DAPE concentrations above 1.5% generally provided good emulsion stability, whereas favorable dosages were approximately 1.0% for L44 and 0.5% for S20. The homogenization times (Table S2) selected for the DAPE, L44, and S20 systems were 5, 5, and 15 min, respectively. Based on these results, the emulsifier dosage ranges were set at 1.4–2.2% for DAPE, 0.6–1.4% for L44, and 0.2–0.6% for S20. The detailed preliminary results are provided in Tables S1 and S2. These ranges were used to establish the three factor levels for the subsequent L9(3^3^) orthogonal experiments. And an emulsifier-free control (0#) was prepared using the same base formulation as the baseline sizing, with the emulsifier omitted.

### 2.3. Three-Factor, Three-Level Orthogonal Design

Based on the preliminary emulsifier screening and the industrial formulation, the dosage ranges of lubricants M and N were both fixed at 0.6–1.0%. Three dosage levels were selected separately for DAPE, L44, and S20. A three-factor, three-level L9(3^3^) orthogonal design was then conducted independently for each emulsifier series, with emulsifier dosage, lubricant M dosage, and lubricant N dosage as the three factors. The factor levels are listed in Table 2, and the complete formulation matrices are provided in Table S3. Each emulsifier family contained nine formulations, giving 27 orthogonal experiments in total.

### 2.4. Characterization of Sizing Emulsions

#### 2.4.1. Solids Content Measurement

The solids content, defined as the nonvolatile fraction of the sizing emulsion, was determined with reference to GB/T 20623-2006. Approximately 1.0 g of emulsion was spread uniformly in a clean, dry weighing dish and heated in an oven at 150 °C to remove water and volatile components until constant mass was reached. The solids content was calculated using Equation (1).

(1) $$w_{1}=\frac{m_{3}-m_{1}}{m_{2}-m_{1}}\times 100\%$$

where *w*_1_ is the solids content of the emulsion (%); *m*_1_ is the weight of the empty container (g); *m*_2_ is the total weight of the container and the emulsion before dehydration (g); and *m*_3_ is the total mass after drying to constant mass (g).

#### 2.4.2. Viscosity Measurement

Viscosity measurements were conducted with an NDJ-8S digital rotational viscometer (Shanghai Shangpu Instrument Equipment Co., Ltd., Shanghai, China)) at room temperature. The spindle and rotational speed were selected so that the instrument reading remained within 10–90% of its effective range. A 20–25 mL aliquot of sizing emulsion was transferred to the measurement cup for the No. 0# spindle, and the rotational speed was set to 60 rpm, corresponding to a shear rate of 73.38 s^−1^. All formulations were tested in three replicates, and the mean values were recorded.

#### 2.4.3. Particle Size Measurement

Droplet size and size distribution were measured with a Bettersize 3000 Plus laser/image particle-size analyzer ((Dandong Bettersize Instruments Ltd., Dandong, Liaoning, China)). The measurements were carried out over a size range of 0.01–2000 μm, with refractive index and absorption coefficient for the particles defined as 1.52 and 0.1, and the water refractive index as 1.333. Three measurements were performed for each emulsion and averaged.

#### 2.4.4. Centrifugal Stability Measurement

Emulsion stability was assessed from resistance to centrifugation-induced separation. Approximately 12 mL of each sizing agent emulsion was centrifuged at 3000 rpm for 30 min. About 1.0 g of the upper supernatant was then transferred to a weighing dish and dried at 150 °C to constant mass to determine its solids content. The percentage decrease in supernatant solids content after centrifugation was calculated using Equation (2).

(2) $$w_{r\;}=\frac{m_{1}-m_{2}}{m_{1}}\times 100\%$$

where *w*_r_ is the decrease in supernatant solids content after centrifugation (%); *m*_1_ is the solids content of the original emulsion (%); and *m*_2_ is the solids content of the centrifuged supernatant (%).

#### 2.4.5. Surface Tension Measurement

The surface tension was assessed with an optical contact angle meter (Theta Flex, Biolin Scientific Oy, Espoo, Finland), based on the pendant-drop approach. The surface tension was determined from the droplet profile immediately before detachment, when the suspended droplet reached its maximum stable volume. Each formulation was analyzed three times, and the mean value was used.

### 2.5. Three-Parameter Evaluation and Formulation Screening

Surface tension, particle size, and centrifugal stability were used to represent, respectively, wetting behavior, droplet dispersion, and resistance to centrifugation-induced separation. Range analysis based on either surface tension or particle size alone yielded different optimum formulations within the same emulsifier family, indicating that a single physicochemical parameter could not adequately describe the state of this multicomponent sizing system. An empirical three-parameter scoring scheme was therefore established.

The weighting coefficients were assigned empirically according to the relative functional relevance of the three parameters to sizing application. Surface tension was given the highest weight (50%) because it is closely associated with the wetting and spreading behavior of the sizing emulsion on basalt fiber surfaces and thus with initial coating formation. Particle size was assigned a weight of 30% because it reflects the dispersion state of the emulsion and may affect the uniformity of the deposited sizing layer. Centrifugal stability was assigned a weight of 20% as an indicator of resistance to phase separation during storage and handling. This weighting scheme prioritizes wetting and coating behavior while retaining dispersion and stability as complementary criteria.

As shown in Table 3, the data range of each parameter was divided into ten intervals and assigned scores of 100, 90, 80, and so forth; lower surface tension, smaller particle size, and lower solids content decrease received higher scores. The parameter scores were then combined according to their assigned weights to obtain an overall score on a 100-point scale, which was subsequently used for range analysis of the orthogonal experiments. It should be noted that because separate scoring ranges were used for the DAPE, L44, and S20 series, the integrated scores were used only to rank formulations within each emulsifier series and were not intended for direct comparisons across the three series. The final formulations for industrial validation were selected based on the within-series screening results and subsequent experimental evaluation.

### 2.6. Industrial Fiber Drawing and Fiber Characterization

#### 2.6.1. Industrial Fiber Drawing

To further verify the feasibility of the multi-parameter screening infiltration agent and assess the effect of the ratio combination of emulsifier and lubricant on the properties of basalt fibers, six selected formulations were prepared and applied on an industrial BF production line. All samples were produced on the same raw materials and processing. Water-drawn fibers (O-1) and fibers produced with the original industrial sizing (O-2) were included as controls. Fibers produced with optimized formulations were designated RA-1, RA-2, RB-1, RB-2, RC-1, and RC-2.

#### 2.6.2. Characterization Modified Basalt Fibers

Fiber diameter, linear density, single-filament tensile strength, yarn breaking strength and chemical resistance were measured with reference to GB/T 7690.5-2013, GB/T 7690.1-2013, DB51/T 2321-2017, GB/T 7690.3-2013, and GB/T 3857 2017, respectively. Diameters were measured on 25 randomly selected filaments; linear density was measured in triplicate; 15 valid measurements were collected for each single-filament tensile test; and yarn breaking strength was measured ten times per sample.

## 3. Results and Discussion

### 3.1. Effects of Emulsifier–Lubricant on the Properties of Sizing Emulsions

#### 3.1.1. Solids Content of the Sizing Emulsions

**Figure 2:**
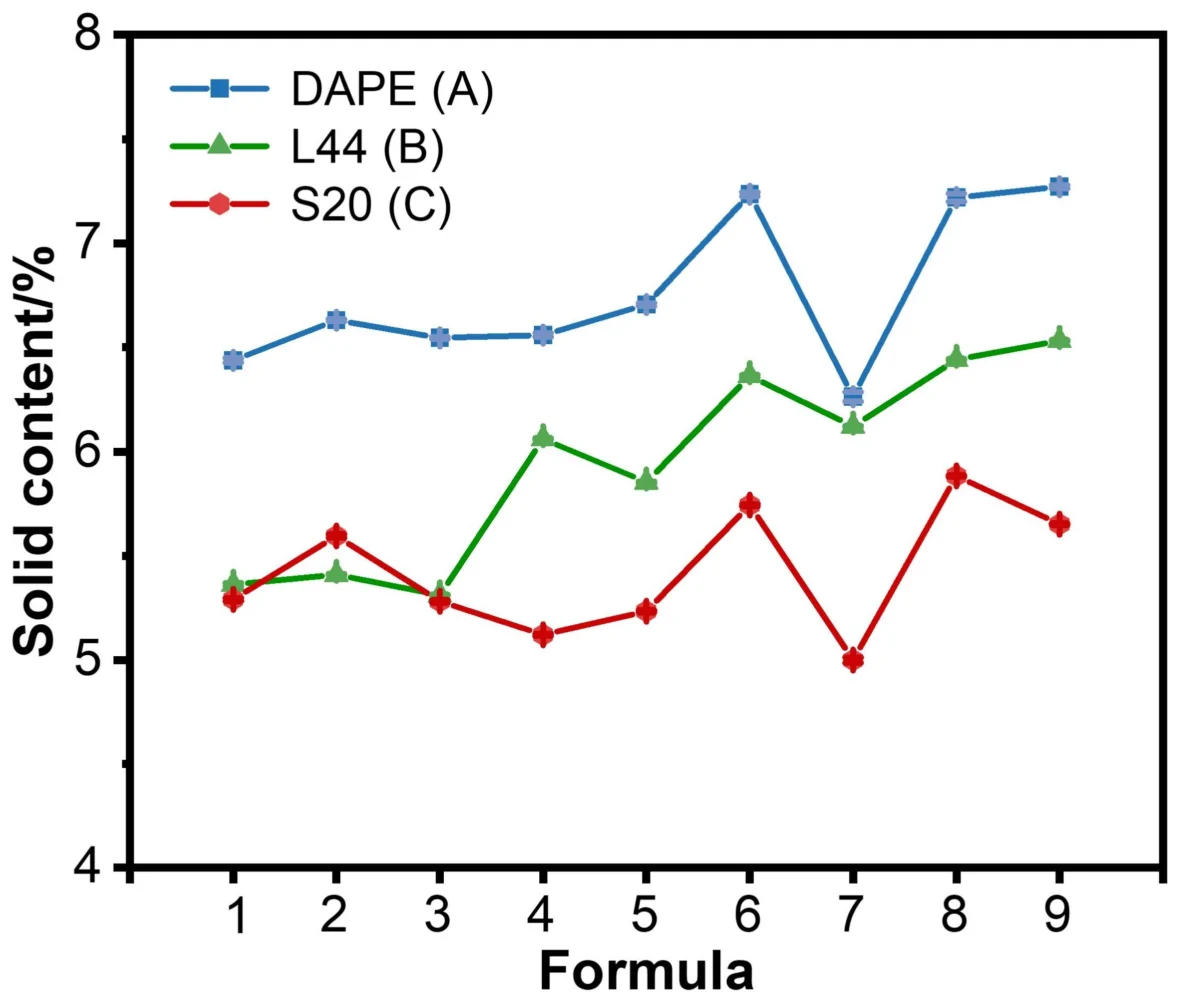
The solids content of the sizing agent in the three series.

Solids content is a basic property of the sizing emulsion and can influence the amount of material deposited on the fiber surface. As shown in Figure 2, the DAPE (A), L44 (B), and S20 (C) formulations all remain within normal ranges. The A, B, and C series gave values of approximately 6.0–7.5%, 5.0–7.0%, and 5.0–6.0%, respectively. The A series generally showed the highest solids content, while the C series showed the lowest. This overall ordering coincided with the combined differences in emulsifier identity, HLB, and dosage among the three series. Although the total amount of the effective components was nearly constant within a given emulsifier series, noticeable differences in measured solids content were observed among emulsifier–lubricant ratios. Such variation may be due to the emulsifier/lubricant ratios influence emulsification, which changes droplet size/distribution and thus packing density, together with electrostatic interactions among dispersed particles [46,47,48].

#### 3.1.2. Viscosity of the Sizing Emulsions

**Figure 3:**
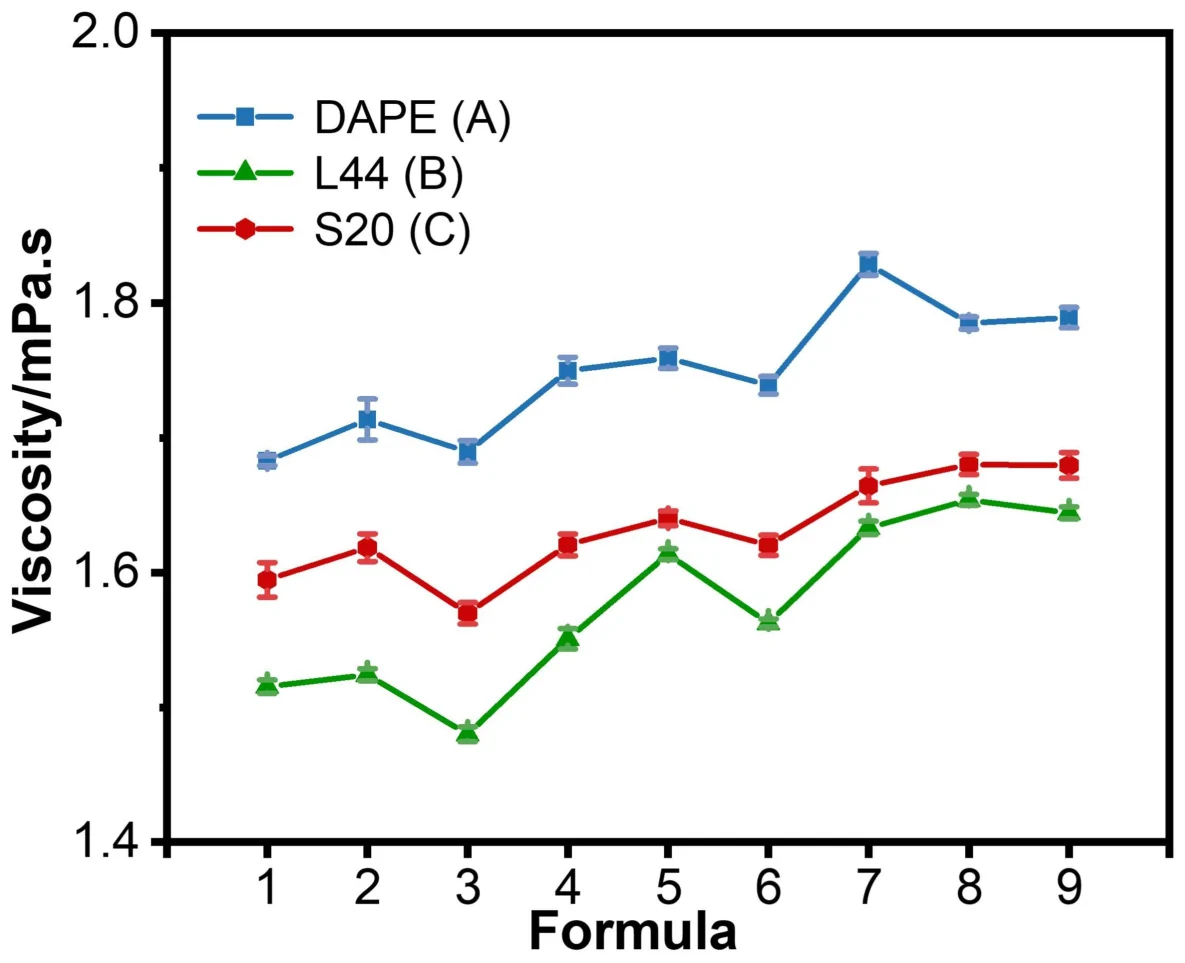
The viscosity of the sizing agent in the three series.

The viscosities of the DAPE (A), L44 (B), and S20 (C) sizing emulsions are presented in Figure 3. Because the formulations contained approximately 92–95% water, they behaved as low-viscosity oil-in-water emulsions, in which the continuous aqueous phase dominated the viscosity of the system. The overall viscosity order was A > B > C, following a positive correlation with the HLB value and dosage of the emulsifier, and solids content. All formulations fell within 1.40–1.90 mPa·s, close to the viscosity of the original industrial sizing, ensuring the normal coating of the emulsion on the fibers.

#### 3.1.3. Particle Size of the Sizing Emulsions

**Figure 4:**
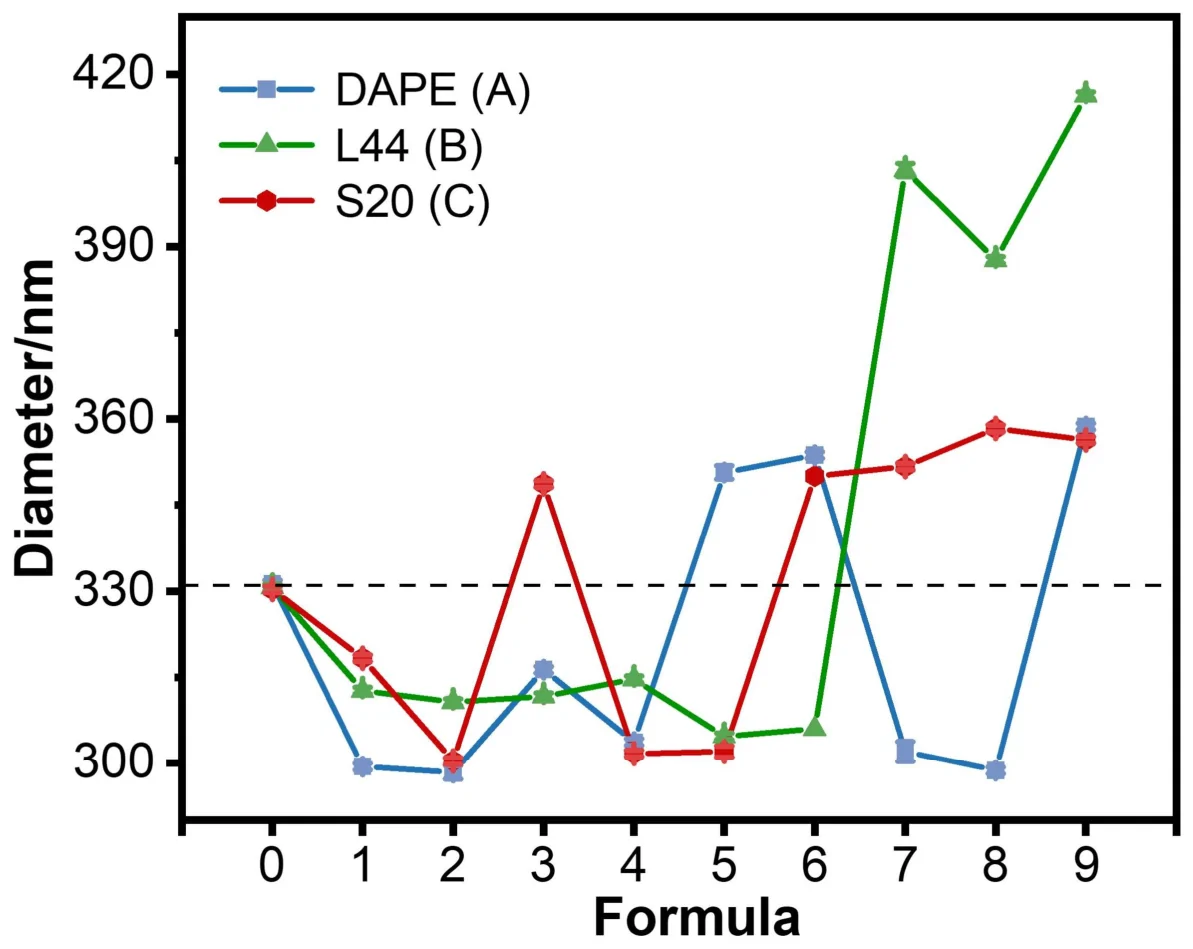
The particle size of the sizing agent in the three series.

Particle diameter and its distribution affect both emulsion stability and deposition of sizing on the filament surface. As shown in Figure 4, increasing DAPE above 1.4% in the A series did not produce a further systematic reduction in particle size, indicating that a higher emulsifier dosage is not necessarily beneficial for dispersion. Five formulations in the B series exhibited larger particle sizes than the emulsifier-free control (0#), suggesting the limited dispersing capability of L44 in this composite formulation. In the C series, particle size remained comparatively low and changed little when S20 was ≤0.4%, while increasing from approximately 310 to 400 nm when the S20 content reached 0.6%. Overall, the three series show that excessive emulsifier dosage or an excessively high total level of emulsifier/lubricant content can increase emulsion particle size.

#### 3.1.4. Centrifugal Stability of the Sizing Emulsions

**Figure 5:**
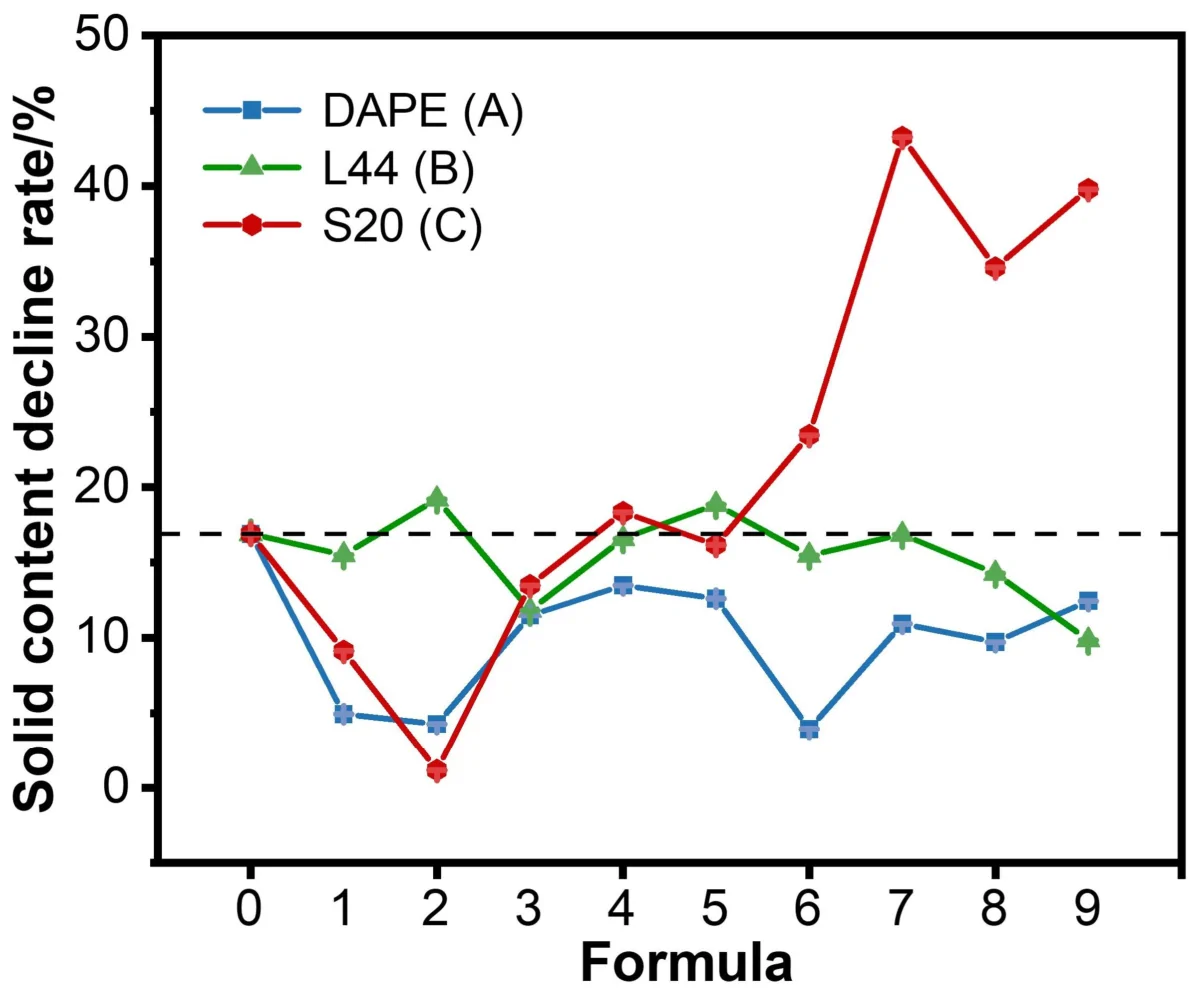
The decrease in supernatant solids content after centrifugation in the three series.

A lower decrease in supernatant solids content after centrifugation indicates higher emulsion stability. As shown in Figure 5, every formulation in the A series exhibited a lower decrease than the emulsifier-free control (0#), indicating that the addition of DAPE favors emulsion stabilization. For series B, five formulations exceeded the control value, and a notable increase when the L44 reached 1.4%, indicating that excessive L44 impaired emulsion stability. At low S20 concentrations, the C series differed only slightly from the emulsifier-free control. These results confirm that the stability of the sizing emulsion depends strongly on the composition and ratio of emulsifiers to lubricants.

#### 3.1.5. Surface Tension of the Sizing Emulsions

**Figure 6:**
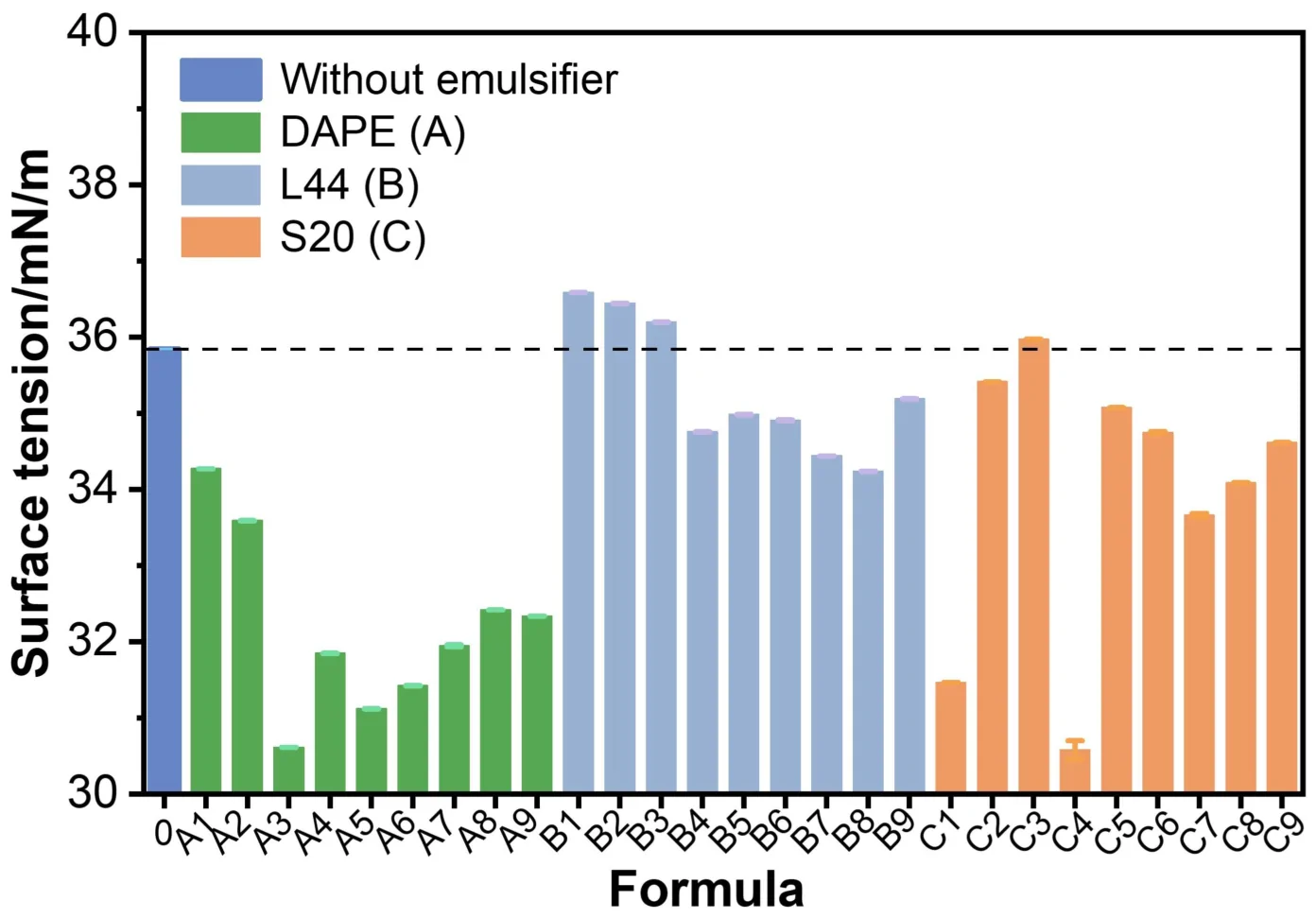
The sizing agent surface tension values in the three series.

Surface tension is closely related to fiber coating and the compatibility with resin matrices. As shown in Figure 6, the emulsifier-free control (0#) had a surface tension of 35.844 mN·m^−1^. All A-series formulations were below this value, with the lowest values occurring overall at 1.8% DAPE. In the B series, low L44 contents produced surface tensions above that of the control, and further addition of L44 did not result in a significant reduction. And most C-series formulations also lowered surface tension, with C3# as the exception. These results suggest that, compared with L44 and S20, the emulsifier DAPE, with its higher HLB value, is more effective in reducing emulsion surface tension, which may be attributed to its stronger hydrophilicity and polyoxymethylene structure [49,50].

### 3.2. Orthogonal Optimization Based on Emulsion Physicochemical Parameters

#### 3.2.1. Single-Parameter Range Analysis

The results of emulsifier dosage ranges and homogenization times are shown in Tables S1 and S2. DAPE above 1.5% gave good emulsion stability, while suitable dosages were about 1.0% for L44 and 0.5% for S20. The selected homogenization times were 5 min for DAPE, 5 min for L44, and 15 min for S20.

Orthogonal range analyses were first performed separately using surface tension and particle size as individual indicators. Based on surface tension, the optimal combinations for series A, B, and C were A_2_M_3_N_3_, B_3_M_2_N_3_, and C_2_M_1_N_1_, respectively (Tables S4–S6). When particle size was used as the sole criterion, the corresponding preferred combinations changed to A_1_M_1_N_1_, B_2_M_2_N_2_, and C_2_M_2_N_1_ (Tables S7–S9). Obviously, the optimum formulation identified from one parameter was not reproduced by the other parameter in any of the three series. A lower surface tension does not necessarily correspond to the smallest particle size, nor does a smaller particle size guarantee greater resistance to phase separation. Therefore, it is necessary to consider wetting, dispersion and stability together.

#### 3.2.2. Three-Parameter Integrated Evaluation

Based on the single-parameter analysis, an empirically weighted comprehensive score was incorporated with surface tension, particle size, and centrifugal stability to rank formulations within each emulsifier series. As seen in Tables S10–S12, the highest-scoring combinations were A_2_M_3_N_3_, B_2_M_2_N_2_, and C_2_M1N_1_ for the A, B, and C series, respectively. In the A series, lubricant N produced the largest range in the integrated score, followed by DAPE, whereas lubricant M had the smallest effect. In the B and C series, the emulsifier factor itself gave the largest range. Furthermore, the integrated optimum coincided with the surface-tension optimum for the A and C series and with the particle-size optimum for the B series.

#### 3.2.3. Selection Formulations

Optimal formulations were selected by considering both the single-parameter analyses (surface tension or particle size) and the three-parameter score. In the A series, the particle-size optimum A_1_M_1_N_1_ had an integrated score of only 46.68, markedly below that of formulation #3 (70.93), and was therefore replaced by A_1_M_3_N_3_. Based on the above results, two formulations from each emulsifier series were ultimately selected for further evaluation. As shown in Table 4, RA-1, RB-2, and RC-1 were identified as the preferred formulations within the A, B, and C series, respectively, and their integrated scores were higher than those of the other tested combinations in the corresponding orthogonal arrays. These results suggest that the use of the surface-tension/particle-size/stability score can serve as a basic intra-series ranking aid within a given emulsifier series.

### 3.3. Performance of Basalt Fibers Treated with Selected Sizing Formulations

To evaluate the practical application of the selected sizing agents and determine how emulsifier–lubricant ratio affected fiber performance, the six selected formulations were prepared and applied during industrial BF drawing under identical production conditions. Water and the original industrial sizing were used as controls. The resulting fibers were characterized by basic physical properties, mechanical performance, and resistance to acid and alkali exposure.

#### 3.3.1. Fiber Morphology and Basic Properties

**Figure 7:**
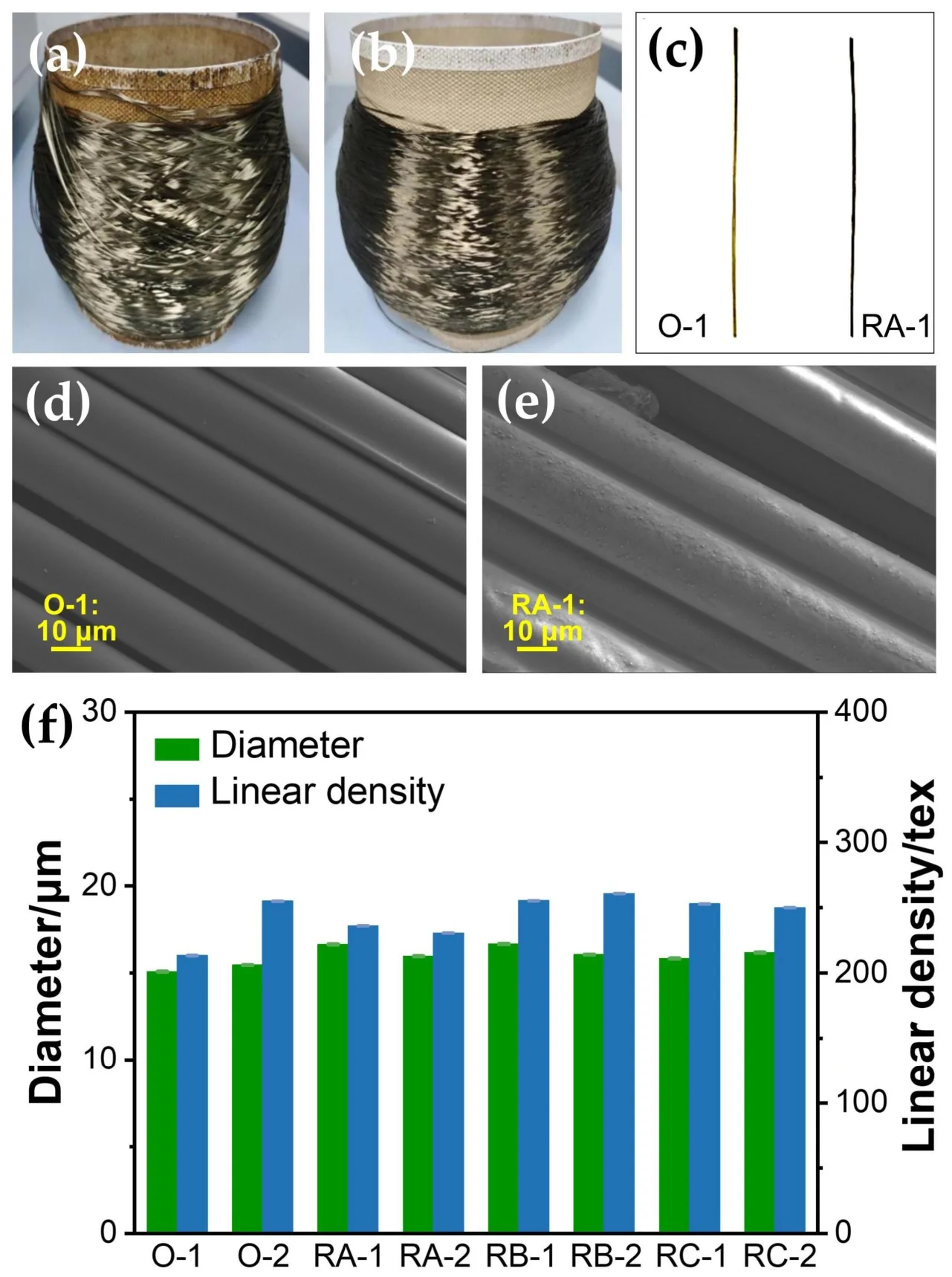
Continuous basalt fibers: (**a**–**c**) macroscopic appearance, (**d**,**e**) SEM images, (**f**) filament diameter, and inear density.

Figure 7a–e shows the surface and microscopic morphologies of O-1 (water-drawn) and RA-1 (sized) fibers. The O-1 filaments exhibited a relatively loose arrangement and poor bundling behavior, whereas RA-1 formed a more compact and continuous fiber strand. SEM images further revealed a smooth and clearly separated filament surface for O-1, while RA-1 was covered by a particulate coating that connected neighboring filaments and increased surface texture. Such coating can provide mechanical protection and strand cohesion during processing.

As shown in Figure 7f, all fibers exhibited a similar nominal filament diameter of approximately 17 μm, and the measured diameters ranged from 15.00 to 17.00 μm. The diameter and linear density values are presented as mean ± standard deviation based on repeated measurements. Among all samples, O-1 exhibited the smallest average diameter (15.08 μm), while the sized fibers showed slightly higher diameters, which may be attributed to the presence of a sizing layer on the filament surface. Correspondingly, O-1 exhibited a relatively lower linear density, which was associated with its smaller filament diameter and the absence of a surface sizing coating.

#### 3.3.2. Mechanical Properties

**Figure 8:**
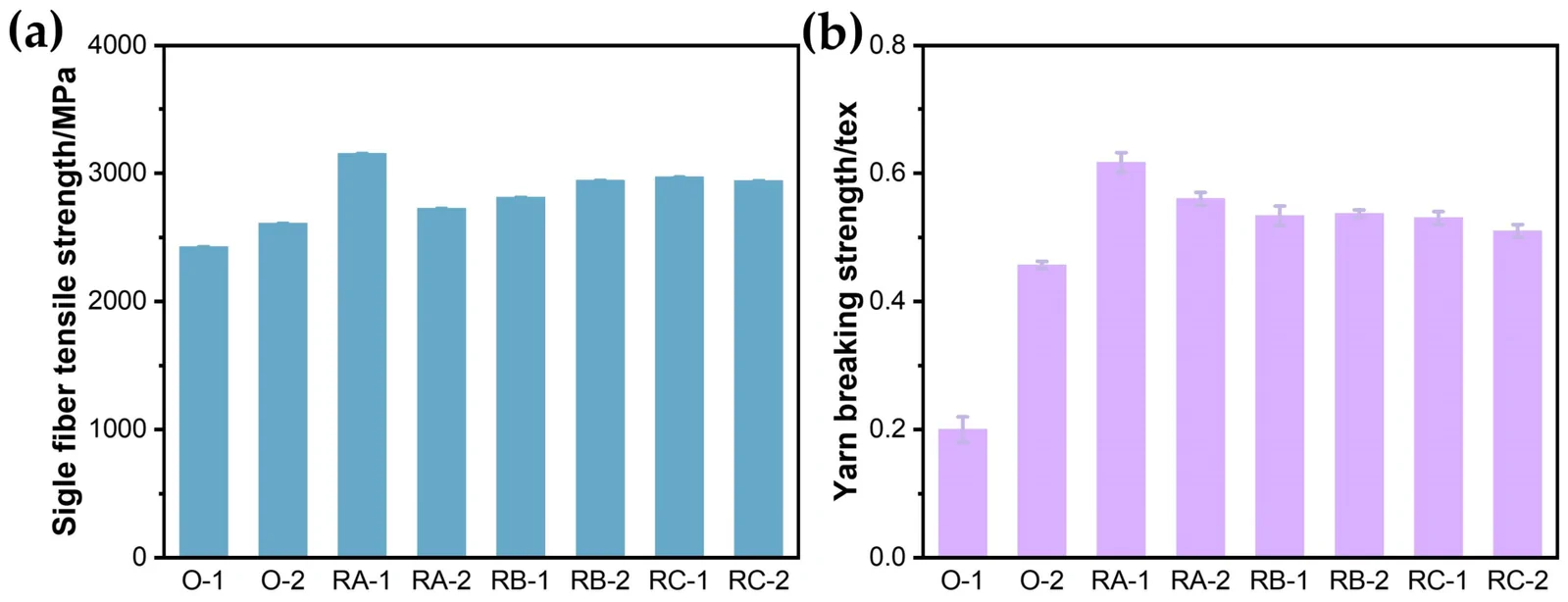
Mechanical properties of basalt fibers produced different sizing formulations: (**a**) single-filament tensile strength and (**b**) yarn breaking strength.

Figure 8a summarizes the single-filament tensile strengths of the eight BF samples. The water-drawn fibers (O-1) and fibers produced using the original industrial sizing agent (O-2) exhibited tensile strengths of 2426.67 and 2608.97 MPa, respectively, showing that the original industrial sizing already improved the strength of the water-drawn fibers. The six selected sizing formulations produced single-filament strengths of 2725.24–3152.21 MPa, corresponding to increases of 12.30–29.90% relative to O-1 and 4.46–20.82% relative to O-2.

Among the tested formulations, RA-1 exhibited the highest tensile strength (3152.21 MPa). The relationship between the integrated evaluation scores and single-filament tensile strength was also examined within the selected formulation pairs. RA-1 (84.98) exhibited higher tensile strength than RA-2 (71.58), while RB-2 (75.79) outperformed RB-1 (70.78), and the same trend was observed for the C series, consistent with the relative ordering of their integrated scores. Within the A series, RA-1 and RA-2 differed only in the emulsifier A content (1.8% versus 1.6%), yet their tensile strength improvements relative to O-2 were 20.82% and 4.46%, respectively. In contrast, the difference in single-filament tensile strength between RC-1 and RC-2 was relatively small. These observations suggest that emulsifier content and its balance with the lubricants may influence sizing performance and the resulting fiber strength. Therefore, given that only six of the 27 formulations underwent industrial fiber-drawing tests, the proposed evaluation system is currently only a basic intra-series ranking aid, and its predictive capability requires further validation using a broader range of formulations.

Yarn breaking strength exhibited a similar trend to that observed for single-filament tensile strength. As shown in Figure 8b, O-1 and O-2 exhibited yarn breaking strengths of 0.20 and 0.46 N·tex^−1^, respectively, whereas the six optimized sizing formulations showed higher values ranging from 0.51 to 0.62 N·tex^−1^, corresponding to improvements of 10.87–34.78% compared with O-2. Among all formulations, RA-1 achieved the highest yarn breaking strength (0.62 N·tex^−1^). Both the single-filament and strand tests consistently identified RA-1 as having the best mechanical performance, indicating that the synergistic combination of DAPE 1.8%/M 1.0%/N 0.6% provided the most favorable balance, within the present baseline formulation, among emulsion wetting, dispersion/stability, and lubrication during fiber drawing.

#### 3.3.3. Chemical Stability

**Figure 9:**
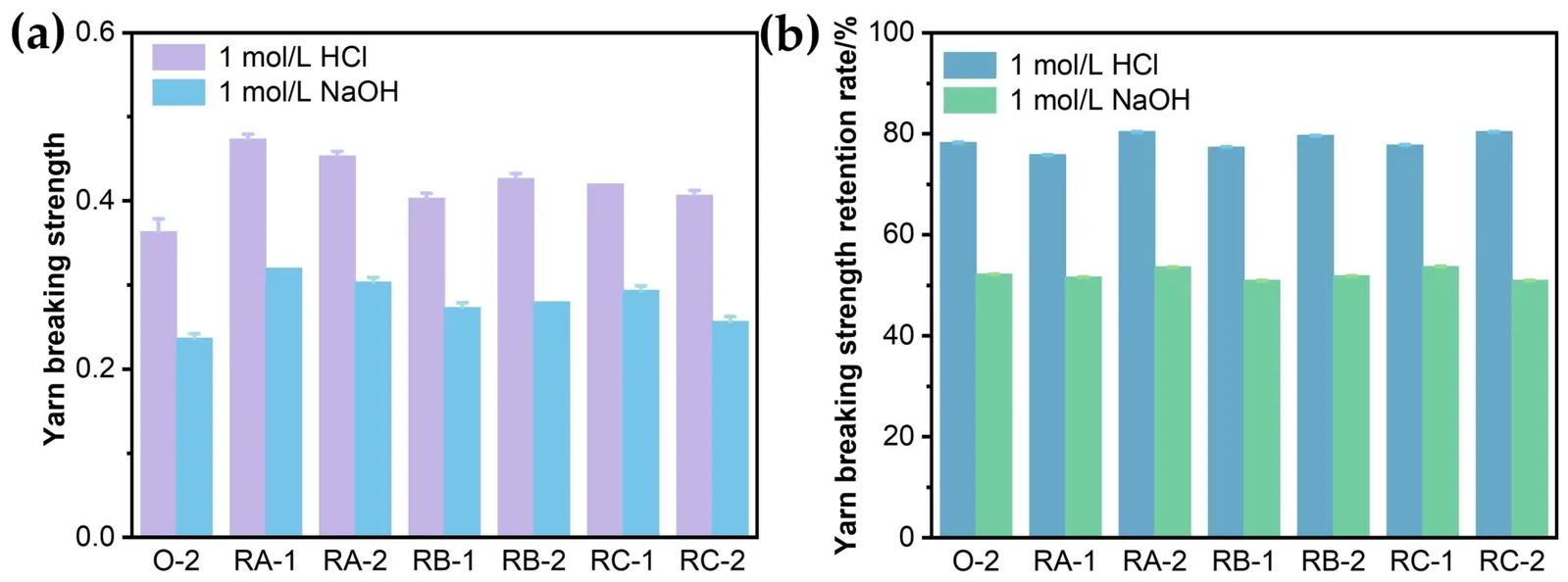
Acid and alkali resistance of basalt fibers are produced with different sizing formulations: (**a**) Yarn breaking strength and (**b**) yarn breaking strength retention rate.

Acid and alkali exposure tests were conducted to evaluate whether the optimized emulsifier–lubricant systems could improve mechanical performance while maintaining the corrosion resistance of basalt fibers. The yarn breaking strengths after treatment with 1 mol/L HCl and 1 mol/L NaOH are summarized in Figure 9.

Water-drawn basalt fibers lack a surface coating, and they entangle after acid treatment and cannot be separated into bundles, making yarn breaking strength difficult to determine. For the remaining seven samples, the yarn breaking strengths after acid treatment ranged from 0.40 to 0.50 N·tex^−1^, values that were 13.89–30.56% higher than those of O-2 (0.36 N·tex^−1^). The corresponding strength retention rates ranged from 75.81% to 80.39%, showing only minor variations among different sizing formulations. These results indicate that optimized sizing formulations improved the absolute mechanical strength retained after acid exposure, while their effect on relative strength retention was limited.

After alkali treatment, O-1 also failed to form suitable bundles for mechanical testing due to severe filament entanglement. The remaining seven samples exhibited greater mechanical loss after exposure to 1 mol/L NaOH, with strength retention rates ranging from 50.94% to 53.70%, considerably lower than those after HCl treatment. The low strength retention after NaOH exposure indicates substantial mechanical degradation of the sized fiber system under the tested alkaline conditions, which is likely attributable to hydrolysis of the unsaturated polyester film-forming layer in NaOH solution as well as to changes in the sizing layer, basalt fiber surface, or fiber sizing interface, thereby leading to the poor alkali resistance of the basalt fibers. Among the seven sizing formulations, only minor variations in strength retention and mass loss were observed after NaOH exposure.

Overall, the optimized sizing formulations improved the initial mechanical performance of the basalt fibers, resulting in higher absolute yarn breaking strengths after acid exposure than that of O-2. However, the relative strength retention rates varied only slightly among formulations. Under alkaline conditions, all seven testable samples exhibited substantial strength loss, with retention rates of 50.94–53.70% and no significant differences among formulations. These results indicate that the current sizing formulations improved the absolute mechanical performance retained after chemical exposure but provided limited improvement in relative strength retention, particularly under strongly alkaline conditions.

## 4. Conclusions

An industrial BF sizing formulation was used to examine how DAPE, L44, and S20 and their ratios with lubricants M and N affect sizing emulsion behavior and fiber performance. A three-parameter comprehensive evaluation method incorporating surface tension, droplet size, and stability was established. The results showed that the emulsifier type, dosage, and emulsifier-to-lubricant ratio jointly influenced the physicochemical properties of the emulsion. Industrial validation showed that the six selected formulations increased single-filament tensile strength by 4.46–20.82% and yarn breaking strength by 10.87–34.78% relative to the original industrial sizing. Among these six formulations, RA-1 exhibited the best overall mechanical performance based on the measured fiber properties. The superior performance of RA-1 originates from the HLB contrast between DAPE (16–17) and the lubricants (≈12). The more hydrophilic DAPE preferentially occupies the oil/water interface, forming a denser hydrated interfacial layer that sterically stabilizes the lubricant droplets, suppresses lubricant migration during drawing, and promotes a more uniform sizing film. This is consistent with the low surface tension (30.552 mN·m^−1^), small particle size (298 nm), and low centrifugal solids decrease (4.103%) of RA-1, and with the compact strand and particulate coating observed by SEM. Within each emulsifier series, higher integrated scores generally coincided with better fiber mechanical performance, suggesting that the integrated score may serve as a basic intra-series ranking aid. However, alkaline exposure caused substantial mechanical degradation, with strength retention rates of only 50.94–53.70% among the tested samples, highlighting the limited alkali resistance of the current sizing system.

## Figures and Tables

**Table 1:** The main constituents of the industrial sizing formulation.

Component	Material	Content	HLB
Film former	Unsaturated polyester resin	7.5%	/
Auxiliary film former	Modified unsaturated epoxy resin	0.6%	/
Coupling agent	γ-Methacryloxypropyltrimethoxysilane (KH570)	0.6%	/
Lubricant M	Saturated-fatty-acid polyoxyethylene ester	0.8%	12
Lubricant N	Unsaturated-fatty-acid polyoxyethylene ester	0.8%	12
Emulsifier	Poly(ethylene glycol) dibasic acid diester (DAPE)	1.8%	16–17

**Table 2:** The factor-level combinations used in the orthogonal experiments.

Factor		Range	Levels		
			Level 1	Level 2	Level 3
Emulsifier	A: DAPE (%)	1.4–2.2	1.4	1.8	2.2
	B: L44 (%)	0.6–1.4	0.6	1.0	1.4
	C: S20 (%)	0.2–0.6	0.2	0.4	0.6
Lubricant M (%)		0.6–1.0	0.6	0.8	1.0
Lubricant N (%)		0.6–1.0	1.0	0.8	0.6

**Table 3:** The data ranges and weights used in the three-parameter evaluation.

Parameter	Data Range			Scoring Scale	Weight (%)
	A	B	C		
Surface tension (mN·m^−1^)	30–35	30–40	30–40	100-point scale	50
Particle size (nm)	280–380	300–400	300–420		30
Centrifugal stability (%)	0–20	0–50	0–20		20

**Table 4:** The composition and physicochemical properties of the selected sizing formulations.

Code	Combination	Emulsifier (%)	M (%)	N (%)	Surface Tension (mN·m^−1)^	Particle Size (nm)	Solids Content Decrease (%)	Score
RA-1	A_2_M_3_N_3_	1.8	1.0	0.6	30.552	298	4.103	84.98
RA-2	A_1_M_3_N_3_	1.6	1.0	0.6	30.613	316	11.487	71.58
RB-1	B_3_M_2_N_3_	1.4	0.8	0.6	33.982	305	19.536	70.78
RB-2	B_2_M_2_N_2_	1.0	0.8	0.8	34.597	300	2.658	75.95
RC-1	C_2_M_1_N_1_	0.4	0.6	1.0	30.550	305	9.537	86.46
RC-2	C_2_M_2_N_1_	0.4	0.8	1.0	31.987	303	10.269	79.05

## Data Availability

The data presented in this study are available on request from the corresponding author.

## Supplementary Materials

The following supporting information can be downloaded at https://www.mdpi.com/article/10.3390/polym18212645/s1, Table S1: Stability of sizing emulsions formulated with different emulsifiers; Table S2: Effect of homogenization time on sizing emulsion properties; Table S3: Orthogonal experimental design; Table S4: Range analysis results of surface tension for sizing emulsions in Series A; Table S5: Range analysis results of surface tension for sizing emulsions in Series B; Table S6: Range analysis results of surface tension for sizing emulsions in Series C; Table S7: Range analysis results of particle size for sizing emulsions in Series A; Table S8: Range analysis results of particle size for sizing emulsions in Series B; Table S9: Range analysis results of particle size for sizing emulsions in Series C; Table S10: Range analysis results of comprehensive scores for sizing emulsions in Series A; Table S11: Range analysis results of comprehensive scores for sizing emulsions in Series B; Table S12: Range analysis results of comprehensive scores for sizing emulsions in Series C.
