# Supplementary material for: Effects of Emulsifier and Lubricant Formulation on Basalt Fiber Sizing Emulsions and Fiber Performance
Source: Polymers (Basel). 2026 Oct 25;18(21):2645. doi: 10.3390/polym18212645 (PMC13765649; doi:10.3390/polym18212645)
Supplement: Supplementary file 1 [file polymers-18-02645-s001.zip › polymers-4561953-Supplementary Material.pdf]

polymers

MDPI

Supporting information

Effects of Emulsifier and Lubricant Formulation on Basalt Fiber Sizing Emulsions and Fiber Performance

Jiajia Yuan [1], Junlan Li [1], Simin Zhuo [2], Jiahao Wu [1], Jianhui Wu [1], Like Zou [2], Yuanfang Deng [3] and Chuan Lai [1,2,*]

[1] Basalt Fiber and Composite Key Laboratory of Sichuan Province, School of Modern Industry for Basalt Fiber Composite Materials, Dazhou Key Laboratory of Advanced Technology for Fiber Materials, Sichuan University of Arts and Science, Dazhou 635000, China
[2] School of Chemistry and Environmental Engineering, Sichuan University of Science and Engineering, Zigong 643000, China
[3] Dazhou Market Supervision and Administration Bureau, Dazhou 635000, China
* Correspondence: laichuanemail@163.com

Table S1. Stability of sizing emulsions formulated with different emulsifiers.

Type	Proportion (%)	Stability evaluation
DAPE	2.0	negligible precipitate, good stability
	1.5	negligible precipitate, good stability
	1.0	slight precipitate, fair stability
	0.5	precipitate formed, moderate stability
L44	2.0	significant precipitate, poor stability
	1.5	precipitate formed, moderate stability
	1.0	slight precipitate, fair stability
	0.5	precipitate formed, moderate stability
S20	2.0	significant precipitate, very poor stability
	1.5	significant precipitate, very poor stability
	1.0	significant precipitate, poor stability
	0.5	slight precipitate, fair stability

Table S2. Effect of homogenization time on sizing emulsion properties.

Type	Parameter	Homogenization time (min)			
		**0**	**5**	**15**	**30**
DAPE	particle size (nm)	304	303	303	303
	solid content reduction rate (%)	3.932	3.679	4.683	3.913
L44	particle size (nm)	352	351	353	354
	solid content reduction rate (%)	34.782	34.504	38.483	35.619
S20	particle size (nm)	305	302	301	303
	solid content reduction rate (%)	17.120	14.565	13.471	18.875

Table S3. Orthogonal experimental design.

**Experiment**		Emulsifier **(%)**		**Lubricant M (%)**		**Lubricant N (%)**	
A series (DAPE)	1	1	1.4	1	0.6	1	1.0
	2	1	1.4	2	0.8	2	0.8
	3	1	1.4	3	1.0	3	0.6
	4	2	1.8	1	0.6	2	0.8
	5	2	1.8	2	0.8	3	0.6
	6	2	1.8	3	1.0	1	1.0
	7	3	2.2	1	0.6	3	0.6
	8	3	2.2	2	0.8	1	1.0
	9	3	2.2	3	1.0	2	0.8
B series (L44)	10	1	0.6	1	0.6	1	1.0
	11	1	0.6	2	0.8	2	0.8
	12	1	0.6	3	1.0	3	0.6
	13	2	1.0	1	0.6	2	0.8
	14	2	1.0	2	0.8	3	0.6
	15	2	1.0	3	1.0	1	1.0
	16	3	1.4	1	0.6	3	0.6
	17	3	1.4	2	0.8	1	1.0
	18	3	1.4	3	1.0	2	0.8
C series (S20)	19	1	0.2	1	0.6	1	1.0
	20	1	0.2	2	0.8	2	0.8
	21	1	0.2	3	1.0	3	0.6
	22	2	0.4	1	0.6	2	0.8
	23	2	0.4	2	0.8	3	0.6
	24	2	0.4	3	1.0	1	1.0
	25	3	0.6	1	0.6	3	0.6
	26	3	0.6	2	0.8	1	1.0
	27	3	0.6	3	1.0	2	0.8

Table S4. Range analysis results of surface tension for sizing emulsions in Series A.

**Statistical item**	**DAPE**	**Lubricant M**	**Lubricant N**	**Blank**
Mean value 1	32.823	32.688	32.701	32.573
Mean value 2	31.461	32.373	32.588	32.319
Mean value 3	32.232	31.455	31.227	31.624
Range value	1.362	1.233	1.474	0.949

Table S5. Range analysis results of surface tension for sizing emulsions in Series B.

**Statistical item**	**L44**	**Lubricant M**	**Lubricant N**	**Blank**
Mean value 1	36.406	35.258	35.240	35.583
Mean value 2	34.878	35.220	35.461	35.260
Mean value 3	34.621	35.427	35.204	35.062
Range value	1.785	0.207	0.257	0.521

Table S6. Range analysis results of surface tension for sizing emulsions in Series C.

**Statistical item**	**S20**	**Lubricant M**	**Lubricant N**	**Blank**
Mean value 1	34.281	31.915	33.430	33.717
Mean value 2	33.486	34.859	33.558	34.598
Mean value 3	34.115	35.108	34.894	33.568
Range value	0.795	3.193	1.464	1.030

Table S7. Range analysis results of particle size for sizing emulsions in Series A.

Statistical item	DAPE	Lubricant M	Lubricant N	Blank
Mean value 1	304.667	302.000	317.000	336.000
Mean value 2	335.667	316.000	320.667	318.333
Mean value 3	320.333	342.667	323.000	306.333
Range value	31.000	40.667	6.000	29.667

Table S8. Range analysis results of particle size for sizing emulsions in Series B.

Statistical item	L44	Lubricant M	Lubricant N	Blank
Mean value 1	323.000	324.333	342.333	325.667
Mean value 2	318.000	320.333	319.667	334.333
Mean value 3	355.333	351.667	334.333	336.333
Range value	37.333	31.334	22.666	10.666

Table S9. Range analysis results of particle size for sizing emulsions in Series C.

Statistical item	S20	Lubricant M	Lubricant N	Blank
Mean value 1	312.000	344.000	335.333	344.667
Mean value 2	308.667	334.333	347.333	340.333
Mean value 3	402.333	344.667	340.333	338.000
Range value	93.666	10.334	12.000	6.667

Table S10. Range analysis results of comprehensive scores for sizing emulsions in Series A.

Statistical item	DAPE	Lubricant M	Lubricant N	Blank
Mean value 1	57.470	56.737	55.690	47.480
Mean value 2	58.680	56.610	51.850	58.917
Mean value 3	54.547	57.350	63.157	64.300
Range value	4.133	0.740	11.307	16.820

Table S11. Range analysis results of comprehensive scores for sizing emulsions in Series B.

Statistical item	L44	Lubricant M	Lubricant N	Blank
Mean value 1	57.900	56.980	52.137	55.703
Mean value 2	62.480	60.877	58.893	54.350
Mean value 3	44.610	47.133	53.960	54.937
Range value	17.870	13.744	6.756	1.353

Table S12. Range analysis results of comprehensive scores for sizing emulsions in Series C.

Statistical item	S20	Lubricant M	Lubricant N	Blank
Mean value 1	60.083	63.103	58.927	55.507
Mean value 2	63.427	49.670	55.167	49.750
Mean value 3	40.180	50.917	49.597	58.433
Range value	23.247	13.433	9.330	8.683
